# Suppression of *Cutibacterium acnes*-Mediated Inflammatory Reactions by Fibroblast Growth Factor 21 in Skin

**DOI:** 10.3390/ijms23073589

**Published:** 2022-03-25

**Authors:** Ying Yu, Yingjie Shen, Siyi Zhang, Nan Wang, Lan Luo, Xinyi Zhu, Xiejun Xu, Weitao Cong, Litai Jin, Zhongxin Zhu

**Affiliations:** School of Pharmaceutical Sciences, Wenzhou Medical University, Wenzhou 325015, China; yuwinn@wmu.edu.cn (Y.Y.); syj583902539@wmu.edu.cn (Y.S.); zsy19970715@wmu.edu.cn (S.Z.); wn18815138519@wmu.edu.cn (N.W.); 1051966855@wmu.edu.cn (L.L.); zhuxinyi09@wmu.edu.cn (X.Z.); ao-yamasan_xu@wmu.edu.cn (X.X.); cwt97126@wmu.edu.cn (W.C.)

**Keywords:** *Cutibacterium acnes*, fibroblast growth factor 21, skin inflammation, keratinocyte-fibroblast cross talk

## Abstract

*Cutibacterium acnes* (*C. acnes*) is a common commensal bacterium that is closely associated with the pathogenesis of acne. Fibroblast growth factor 21 (FGF21), as a favorable regulator of glucose and lipid metabolism and insulin sensitivity, was recently shown to exert anti-inflammatory effects. The role and mechanism of FGF21 in the inflammatory reactions induced by *C. acnes*, however, have not been determined. The present study shows that FGF21 in the dermis inhibits epidermal *C. acnes*-induced inflammation in a paracrine manner while it functions on the epidermal layer through a receptor complex consisting of FGF receptor 1 (FGFR1) and β-Klotho (KLB). The effects of FGF21 in heat-killed *C. acnes*-induced HaCaT cells and living *C. acnes*-injected mouse ears were examined. In the presence of *C. acnes*, FGF21 largely counteracted the activation of Toll-like receptor 2 (TLR2), the downstream nuclear factor-κB (NF-κB), and mitogen-activated protein kinase (MAPK) signaling pathways induced by *C. acnes*. FGF21 also significantly reduced the expression of proinflammatory cytokines, including interleukin (IL)-1β, IL-6, IL-8, and tumor necrosis factor (TNF)-α. Taken together, these findings indicate that FGF21 suppresses *C. acnes*-induced inflammation and might be used clinically in the management and treatment of acne.

## 1. Introduction

Acne vulgaris, the eighth-most-prevalent disease globally in 2010, is a common chronic inflammatory skin disorder that often involves the pilosebaceous follicles [1]. Three main factors involved in the pathogenesis of acne have been identified: hyperproliferation and abnormal differentiation of keratinocytes in hair follicle epithelium, excessive sebum production in sebaceous glands, and dysregulation of the hormone microenvironment [2,3,4]. *Cutibacterium acnes* (*C. acnes*) is a Gram-positive anaerobic bacterium that adheres to the hair shafts and the epithelial walls of follicles. *C. acnes* has long been considered the main cause of acne, and it is consistently one of the most abundant genera in persons with acne [5,6]. *C. acnes* has been shown to coexist with other horny bacteria, such as Corynebacteria, and the symbiotic fungus Malassezia in moist skin areas [7]. In addition, *C. acnes* has been shown to form biofilms in hair follicles within sebaceous glands in most patients with acne [8,9,10].

The pleiotropic cellular effects of three members of the endocrine fibroblast growth factor (FGF) family, FGF19, FGF21, and FGF23, are regulated by their combining with and activating a dual complex composed of FGF receptors (FGFR) that bind to either α-Klotho (KLA) or β-Klotho (KLB) receptors [11]. What is more, FGF21 is expressed primarily in the liver and acts as a hormone, regulating various pharmacological activities and systemic energy metabolism, especially glucose and lipid metabolism, and sensitivity to insulin [12,13,14,15]. FGF21 has recently been reported to relieve acute pancreatitis and alter the serum concentrations of interleukin (IL)-6 and tumor necrosis factor (TNF)-α in mice [16]. FGF21 deficiency has been proved to promote the development of inflammation, intrahepatic steatosis, hepatocyte damage, and fibrosis, whereas FGF21 analogs have potentially been introduced to inhibit nonalcoholic steatohepatitis (NASH) by weakening these processes [17]. Although early studies have shown that FGF21 can regulate inflammation in some diseases, its ability to suppress *C. acnes*-induced inflammatory responses during the development of acne remains unknown.

The skin is the largest organ of the body and consists of many types of cells [18]. Keratinocytes are the main cellular component of the epidermis, constituting the first line of defense against skin pathogens and playing an important role in the innate immune system. In response to external stimuli, such as trauma, bacterial and viral infections, and ultraviolet radiation, keratinocytes produce large numbers of cytokines, chemokines, and antimicrobial peptides [19,20,21,22]. During skin infection by *C. acnes*, keratinocytes secrete numerous inflammatory cytokines, including IL-1β, IL-6, IL-8, and TNF-α [23]. The dermis contains many resident immune cells, including dendritic cells and memory T cells. The infiltration of immune cells during inflammatory responses leads to a substantial increase in the number of immune cells in the skin [24]. Epithelial–mesenchymal crossover was found to play a key role in fibroblast connective tissue disease, with epithelial cells activating underlying fibroblasts, contributing to idiopathic pulmonary fibrosis [25,26] and systemic sclerosis [27]. Moreover, re-epithelialization is influenced by interactions between fibroblasts and keratinocytes [28,29].

Toll-like receptors (TLRs) are important sensors of the innate and adaptive immune systems during acute and chronic inflammation and systemic autoimmune diseases [30,31]. Keratinocytes express a variety of TLRs, including TLR2, which plays an important role in the initiation of innate immune responses in the skin [32,33]. TLR2 forms heterodimers with either TLR1 or TLR6 and reacts with lipopeptides from various microorganisms, including Gram-positive bacteria [34]. The binding of specific ligands to TLR2 triggers key signaling pathways involving nuclear factor-κB (NF-κB) and mitogen-activated protein kinase (MAPK) [35,36].

The present study was designed to determine whether FGF21 had effective anti-inflammatory properties in the epidermal layer through the activation of FGFR1 and KLB.

## 2. Results

### 2.1. FGF21 Is Upregulated in C. acnes-Stimulated Skin

**Figure 1:**
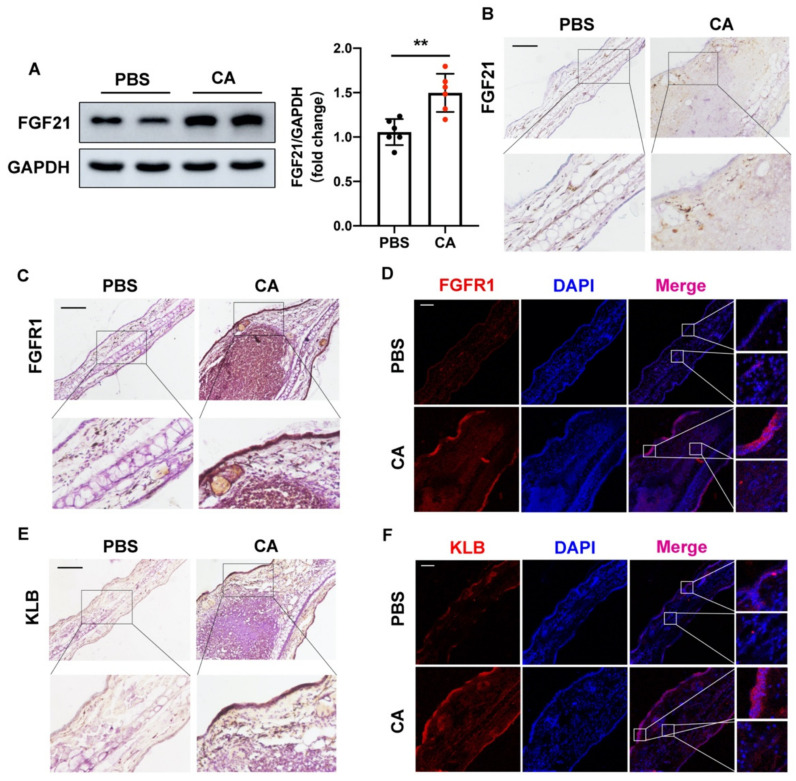
Upregulation of FGF21 in *C. acnes*-stimulated skin. (**A**) Protein expression and quantitative analysis of FGF21 in mouse ears treated with phosphate buffered saline (PBS) or 2 × 10^7^ CFU/mL living *C. acnes* (CA) for 24 h (*n* = 5 mice/group). (**B**,**C**,**E**) Representative IHC staining of FGF21, FGFR1, and KLB in ears of PBS and *C. acnes*-induced mice (*n* = 5 mice/group; magnification ×100). Scale bar = 100 μm. (**D**,**F**) Representative confocal scans are shown for FGFR1 and KLB (red), and the nuclei are stained with DAPI (blue) (*n* = 5 mice/group). The pictures in the upper-right corner and the lower-right corner are enlarged views of the epidermis and the dermis in the box areas, respectively. Scale bar = 100 μm. All data are presented as the mean ± the SD; ** *p* < 0.01.

To analyze the correlation between FGF21 and acne, *C. acnes* was injected intradermally into the ears of mice, which were harvested 24 h later. Western blotting showed that FGF21 protein expression was significantly higher in the ears of C. acnes-treated mice than in the ears of control mice (Figure 1A). Immunohistochemical (IHC) staining also showed that FGF21 expression was obviously increased in ears injected with *C. acnes*. Interestingly, we found that most of the upregulated expression of FGF21 was accumulated in the dermis (Figure 1B). Because FGF21 was shown to act by binding to a receptor complex consisting of FGFR1 and KLB [30], the levels of expression of FGFR1 and KLB in mouse ears were measured. Although the levels of expression of FGFR1 and KLB were low in PBS-injected mice, they were greatly elevated in the epidermis of C. acnes-injected mice. Furthermore, the expression of FGFR1 and KLB was slightly elevated in areas of inflammatory cell infiltration (Figure 1C–F and Figure S1).

Given that FGF21 was mostly secreted by dermal cells during C. acnes-induced inflammation, whereas FGF21 became bound to ligands and receptors that were primarily located in epidermal cells, suggests that the dermal–epithelial crosstalk regulated by FGF21 may be involved in this anti-inflammatory process.

### 2.2. FGF21 Acts in a Paracrine Manner to Exert Anti-Inflammatory Functions in C. acnes-Stimulated Cultured Keratinocytes

**Figure 2:**
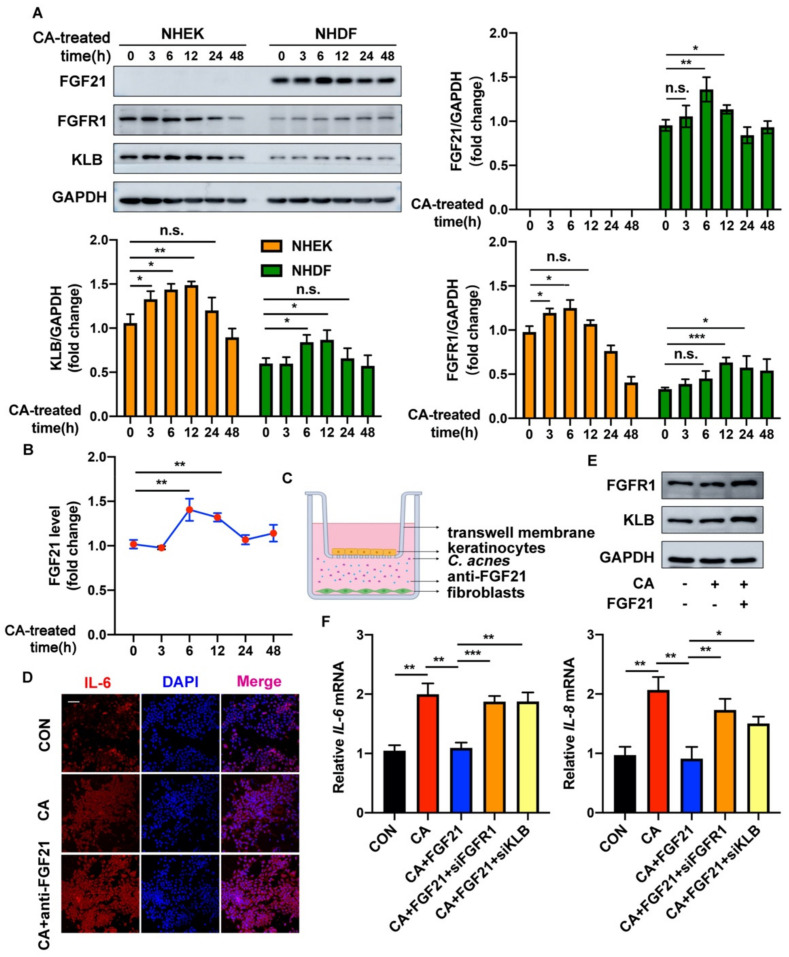
FGF21 acts in a paracrine manner to exert anti-inflammatory functions in *C. acnes*-stimulated cultured keratinocytes. (**A**) Protein expression levels of FGF21 in NHEK and NHDF cells subjected to control or 1 × 10^7^ CFU/mL heat-killed *C. acnes* stimulation at different time points. (**B**) NHDFs were exposed to heat-killed *C. acnes*. The secretion of FGF21 was measured by an ELISA from NHDF culture supernatants. (**C**) Schematic showing an NHEK/NHDF co-culture experiment; FGF21-neutralizing antibody was added after *C. acnes* were stimulated for 6 h in NHDFs. (**D**) Representative images of IL-6 (red)- and DAPI (blue)-stained NHEKs. Scale bar = 100 μm. (**E**) Under the induction of *C. acnes*, the protein expression levels of FGFR1 and KLB with FGF21 treatment in HaCaT cells. (**F**) Quantification of inflammatory cytokine *IL-6* and *IL-8* mRNA levels in different groups in HaCaT cells. All data are presented as the mean ± the SD; * *p* < 0.05, ** *p* < 0.01, and *** *p* < 0.001; n.s., non-significant.

Dermal fibroblasts and epidermal keratinocytes are the major cell types in the skin, with their activities being closely linked. Normal human primary epidermal keratinocytes (NHEKs) and human primary dermal fibroblasts (NHDFs) were therefore isolated, and their activities were further explored. To clarify the modulation pattern of FGF21 in the microenvironment during *C. acnes*-induced inflammation, the levels of expression by these cells of the proteins FGF21, FGFR1, and KLB in response to *C. acnes* were measured at various time points. Relative to the baseline, FGF21 expression was significantly increased 6 h after treatment with heat-killed *C. acnes* in NHDFs. In contrast, FGF21 expression was barely detected at any time point in NHEKs. In addition, *C. acnes* stimulation mainly increased the expression levels of the proteins FGFR1 and KLB in NHEKs (Figure 2A). An enzyme-linked immunosorbent assay (ELISA) showed that the amount of FGF21 in the culture medium of fibroblasts increased significantly after these cells were stimulated with *C. acnes*, with FGF21 concentrations peaking at about 1.4-fold after 6 h (Figure 2B). These findings show that FGF21 is primarily secreted by NHDFs but not by NHEKs.

The effects of FGF21 were further evaluated in co-cultures of fibroblasts and keratinocytes (Figure 2C). Treatment of NHDFs with *C. acnes* resulted in secretion into the medium of factors that inhibited the inflammation of co-cultured NHEKs, as shown by immunofluorescence analysis of IL-6. Treatment with FGF21-neutralizing antibody significantly augmented the expression of IL-6 in keratinocytes (Figure 2D and Figure S2A). To determine whether FGF21 affected the formation of an FGFR1/KLB complex, HaCaT cells were treated with FGF21, significantly enhancing the expression of the proteins FGFR1 and KLB (Figure 2E and Figure S2B). To evaluate the effect of the FGF21/FGFR1/KLB complex on *C. acnes*-induced inflammation, FGFR1-specific or KLB-specific siRNA was transfected into HaCaT cells, increasing the expression of IL-6 and IL-8 mRNAs, a finding indicating that the anti-inflammatory effects of FGF21 were substantially reversed (Figure 2F and Figure S2C). Immunofluorescence staining showed similar results in the case of FGF21 treatment, where blocking of FGFR1 or KLB substantially increased the fluorescence intensity of IL-6 (Figure S2D,E).

Taken together, these findings indicate that FGF21 is secreted by fibroblasts and reduces keratinocyte inflammation in a paracrine manner through the activation of FGFR1 and KLB.

### 2.3. FGF21 Inhibition of the C. acnes-Induced Proinflammatory Response Is Associated with the TLR2/NF-κB/MAPK Signaling Pathways In Vitro

**Figure 3:**
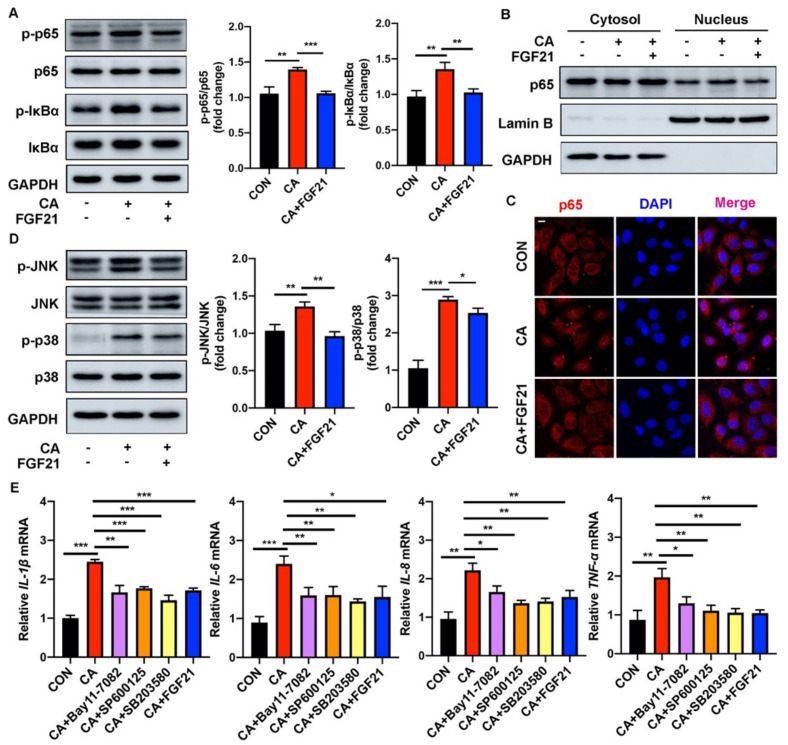
FGF21 suppresses *C. acnes*-induced proinflammatory response by inhibiting NF-κB/MAPK signaling pathways in HaCaT cells. (**A**) Protein expression levels of p65 and IκB in HaCaT cells stimulated with heat-killed *C. acnes* (1 × 10^7^ CFU/mL) for 24 h with and without FGF21 treatment (20 ng/mL). (**B**) The nuclear and cytosolic fractions of p65 assayed by Western blotting. (**C**) Representative confocal scans are shown for p65 (red) and the nuclei are stained with DAPI (blue). Scale bar = 10 μm. (**D**) Protein expression levels of JNK and p38 in heat-killed *C. acnes*-stimulated HaCaT cells with and without FGF21 treatment. (**E**) The mRNA levels of pro-inflammatory factors (*IL-1β*, *IL-6*, *IL-8*, and *TNF-α*) in HaCaT cells, treated with inhibitors of NF-κB (BAY11-7082), JNK (SP600125), p38 (SB203580), or FGF21. All data are presented as the mean ± the SD; * *p* < 0.05, ** *p* < 0.01, and *** *p* < 0.001.

Recombinant human EGF (rhEGF) treatment has been reported to reduce the activities of TLR2 and NF-κB triggered by heat-killed *C. acnes* in a dose-dependent manner. To determine whether FGF21 effectively inhibits the activation of the NF-κB signaling pathway, the phosphorylation of p65 and IκBα in *C. acnes*-treated HaCaT cells was analyzed by Western blotting. *C. acnes* stimulation significantly upregulated the phosphorylation of both p65 and IκBα, whereas FGF21 largely downregulated their phosphorylation (Figure 3A). In addition, the increased nuclear translocation of NF-κB p65 in *C. acnes*-treated HaCaT cells was decreased by FGF21 treatment, a finding consistent with the results of p65 immunofluorescence staining (Figure 3B,C and Figure S3A,B). Because of the close association between the NF-κB and MAPK signaling pathways, the status of two MAPK family members (JNK and p38) was evaluated in these cells. *C. acnes* stimulation enhanced the phosphorylation of JNK and p38, but this activation was significantly ameliorated by FGF21 treatment, indicating that FGF21 inhibited *C. acnes*-induced activation of MAPK signaling in HaCaT cells (Figure 3D). Meanwhile, specific inhibitors of NF-κB, JNK, and p38 as positive controls for FGF21 apparently attenuated the *C. acnes*-induced expression of mRNAs encoding the inflammatory cytokines *IL-1β*, *IL-6*, *IL-8*, and *TNF-α* (Figure 3E).

**Figure 4:**
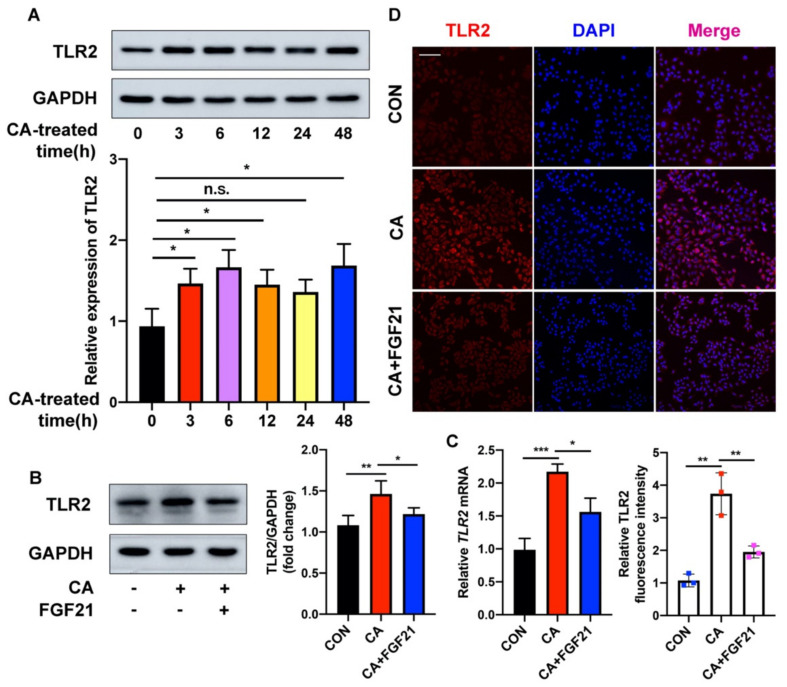
FGF21 ameliorates *C. acnes*-induced TLR2 activities in HaCaT cells. (**A**,**B**) The time points included 0, 3, 6, 12, 24, and 48 h after stimulating with *C. acnes* at the density of 1 × 10^7^ CFU/mL. The protein expression level of TLR2 was detected and analyzed in *C. acnes*-stimulated HaCaT cells with and without FGF21 treatment (20 ng/mL). (**C**) Quantification of *TLR2* mRNA level in HaCaT cells subjected to control, *C. acnes* stimulation, or FGF21 treatment. (**D**) Representative confocal scans are shown for TLR2 (red), and the nuclei are stained with DAPI (blue). Scale bar = 100 μm. All data are presented as the mean ± the SD; * *p* < 0.05, ** *p* < 0.01, and *** *p* < 0.001; n.s., non-significant.

TLR2, a membrane receptor expressed on the surface of certain cells, recognizes foreign substances and delivers appropriate signals to local cells. To explore the underlying mechanism by which FGF21 inhibits *C. acnes*-induced inflammation in HaCaT cells, the modulation of TLR2 in these cells was investigated. *C. acnes* treatment increased the expression of TLR2 protein at 3 h, with the expression of TLR2 peaking at 6 h (Figure 4A). In contrast, FGF21 largely suppressed the *C. acnes*-induced upregulation of TLR2 in HaCaT cells (Figure 4B). The level of *TLR2* mRNA also increased in *C. acnes*-treated HaCaT cells but was decreased by FGF21 treatment (Figure 4C). In addition, immunofluorescence staining showed that the levels of TLR2 were lower in FGF21-treated groups than in control groups (Figure 4D). Taken together, these findings show that FGF21 plays an anti-inflammatory role in *C. acnes* stimulation in vitro.

### 2.4. Anti-Inflammatory Effects of FGF21 on C. acnes Stimulation In Vivo

**Figure 5:**
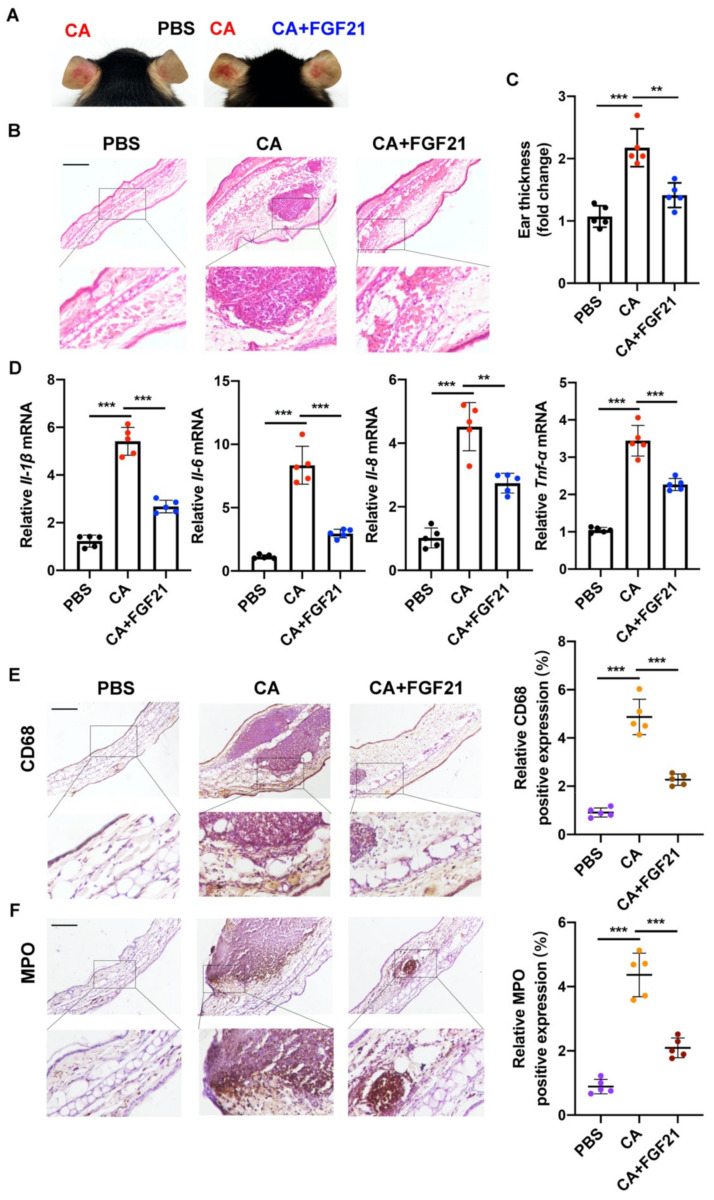
Anti-inflammatory effects of FGF21 against *C. acnes* stimulation in mouse ears. (**A**) FGF21 (50 μg/mL) treatment reduced redness and swelling in the inflamed ears of mice injected with *C. acnes* (2 × 10^7^ CFU/mL) (*n* = 5 mice/group). (**B**) Representative hematoxylin and eosin (HE) staining images from each study group, PBS groups, living *C. acnes*-injected groups, and living *C. acnes*- and FGF21-injected groups (*n* = 5 mice/group). (**C**) Ear thickness changes in *C. acnes*-stimulated mice with and without FGF21 treatment (*n* = 5 mice/group). (**D**) Quantification of pro-inflammatory factor (*Il-1β*, *Il-6*, and *Tnf-α*) mRNA expression in mouse ears subjected to *C. acnes* stimulation with and without FGF21 treatment (*n* = 5 mice/group). (**E**,**F**) Representative IHC staining of MPO and CD68 in mouse ears subjected to *C. acnes* stimulation and FGF21 treatment (*n* = 5 mice/group, magnification ×100). Scale bar = 100 μm. All data are presented as the mean ± the SD; ** *p* < 0.01, and *** *p* < 0.001.

The anti-inflammatory effects of FGF21 on skin injury induced by *C. acnes* were investigated by monitoring the morphological changes in the appearance of mouse ears. Visible histological changes, such as cutaneous erythema and swelling, were observed 24 h after the injection of *C. acnes*, with these alterations alleviated by FGF21 treatment (Figure 5A). In addition, the numbers of infiltrated inflammatory cells and the thickness of mouse ears were markedly increased following *C. acnes* injection, with both significantly reduced by FGF21 treatment (Figure 5B,C). In terms of the cytokines associated with the inflammation after 24 h of *C. acnes* stimulation, the levels of mRNA encoding the pro-inflammatory cytokines of *Il-1β*, *Ιl-6*, *Il-8*, and *Tnf-α* were markedly increased, with all of these levels being effectively reduced by FGF21 treatment (Figure 5D). IHC staining showed that the levels of expression of the neutrophil marker MPO and the macrophage marker CD68 were both markedly enhanced by *C. acnes* stimulation, further indicating that *C. acnes* induces distinct inflammatory responses in mouse ears, whereas FGF21 significantly reduces macrophage and neutrophil infiltration (Figure 5E,F). Taken together, these findings confirm that FGF21 plays a therapeutic regulatory role in inflammation induced by *C. acnes*.

### 2.5. FGF21 Suppresses C. acnes-Induced Inflammation by Inhibiting the TLR2/NF-κB/MAPK Signaling Pathways In Vivo

**Figure 6:**
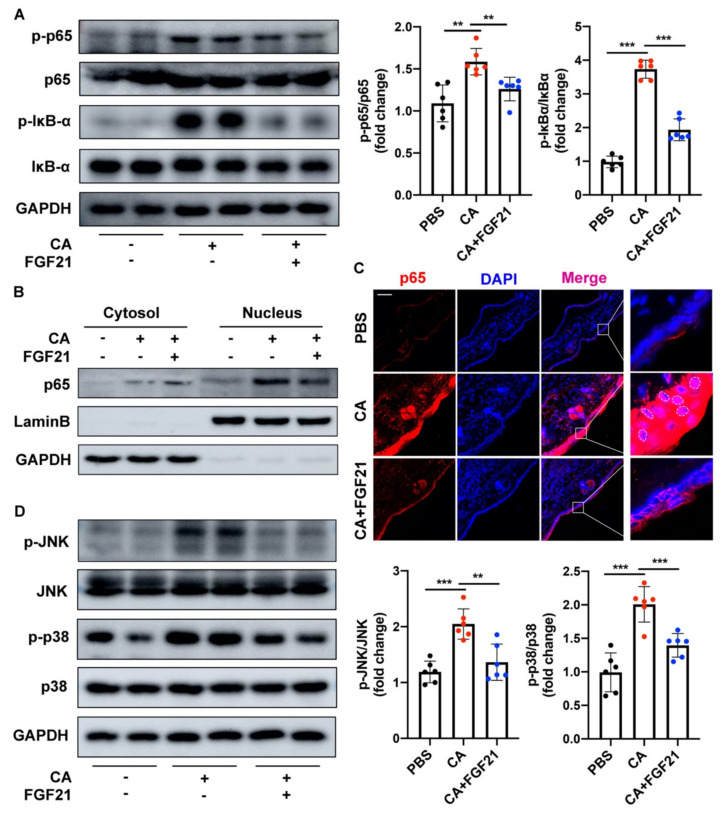
FGF21 suppresses *C. acnes*-induced inflammation by inhibiting TLR2/NF-κB/MAPK signaling pathways in mouse ears. (**A**) Protein expression levels of p65 and IκB in *C. acnes*-stimulated mouse ears with and without FGF21 treatment (*n* = 6 mice/group). (**B**) The nuclear and cytosolic fractions of p65 assayed by Western blotting (*n* = 6 mice/group). (**C**) Representative confocal scans are shown for p65 (red), and the nuclei are stained with DAPI (blue) (*n* = 6 mice/group). Scale bar = 50 μm. (**D**) Protein expression levels of JNK and p38 in *C. acnes*-stimulated mouse ears with and without FGF21 treatment (*n* = 6 mice/group). All data are presented as the mean ± the SD; ** *p* < 0.01, and *** *p* < 0.001.

The mechanism underlying the ability of FGF21 to inhibit the *C. acnes*-induced activation of TLR2-NF-κB/MAPK signaling was further explored in vivo. In parallel, Western blotting showed that *C. acnes* increased the phosphorylation levels of p65 and IκBα but these increases were significantly inhibited by treatment with FGF21 (Figure 6A). The nuclear and cytosolic fractions of mouse ear tissue were subsequently separated, and the levels of p65 in each were assessed. Western blot analysis showed increased nuclear translocation of p65 in *C. acnes*-treated mice, whereas FGF21 inhibited p65 entry into the nucleus and generally redistributed this protein throughout the cytoplasm (Figure 6B and Figure S3C). Consistent with the results obtained in vitro (Figure 3C), *C. acnes*-stimulated mouse ears showed an evident increase in p65 in the epidermis. A large fraction of p65 (dotted circles) entered the nucleus and a portion of p65 was located near infiltrating inflammatory cells, with both being greatly reduced by FGF21 treatment (Figure 6C and Figure S3D). Moreover, *C. acnes* increased the phosphorylation levels of JNK and p38, with both being reduced by FGF21 treatment (Figure 6D).

**Figure 7:**
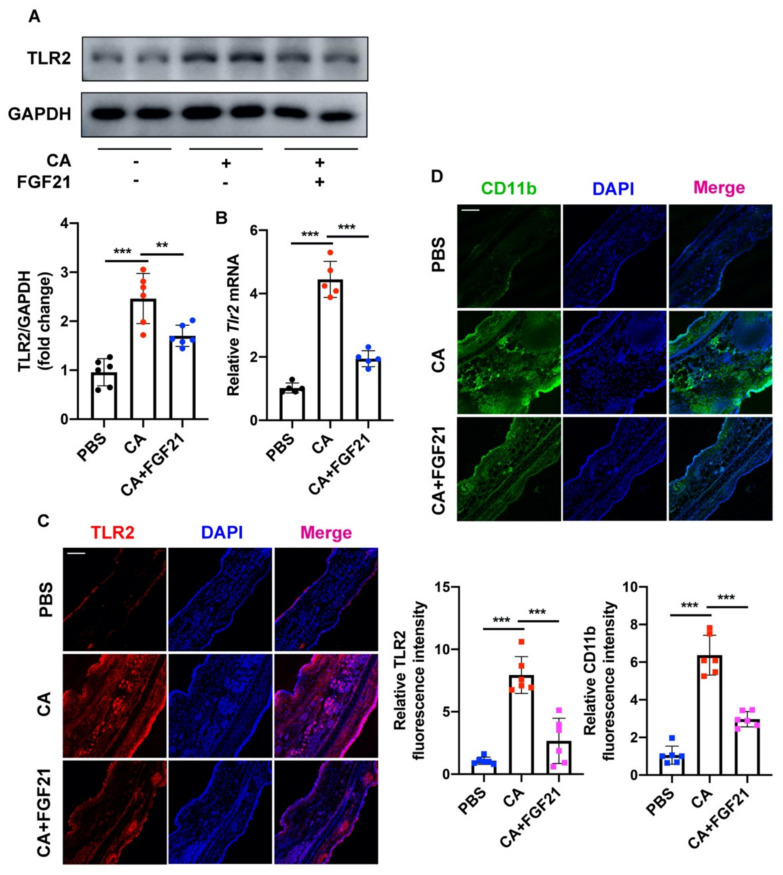
FGF21 alleviation of *C. acnes*-induced TLR2 in mouse ears. (**A**) Protein expression levels of TLR2 in *C. acnes*-stimulated mouse ears with and without FGF21 treatment (*n* = 6 mice/group). (**B**) Quantification of *Tlr2* mRNA level in the ears of mice treated with FGF21 after *C. acnes* stimulation. (*n* = 5 mice/group). (**C**,**D**) Representative confocal scans are shown for TLR2 (red) and CD11b (green), and the nuclei are stained with DAPI (blue) (*n* = 6 mice/group). Scale bar = 100 μm. All data are presented as the mean ± the SD; ** *p* < 0.01, and *** *p* < 0.001.

The in vivo effects of *C. acnes* and FGF21 on TLR2 activation were also assessed in mouse ears. The levels of TLR2 protein and mRNA were both higher in the *C. acnes*-injected groups but largely declined after FGF21 treatment (Figure 7A,B). CD11b regulates several key biological functions of innate immune cells, including the Toll-like receptor signaling pathway, which mediates the production of type I interferon and serves as a marker of inflammatory microenvironments. Evaluation of the fluorescence intensities of TLR2 and CD11b showed that both were significantly enhanced in *C. acnes*-treated mice but these enhancements were lessened by FGF21 treatment (Figure 7C,D).

Taken together, these findings confirm that FGF21 has the anti-inflammatory activities in this model and may prevent pathological changes and mitigate disease development in patients with acne.

## 3. Discussion

Acne vulgaris is an inflammatory skin disease that develops in the sebaceous gland units of the hair follicles, which are colonized by *C. acnes* [37,38]. The pathogenesis of acne involves many processes, such as excessive sebaceous glands and hyperkeratosis of the funnels of hair follicles. Moreover, cysts, papules, pustules, nodules, and comedones were found to be manifestations of acne [39]. Acne has marked psychological, economic, and clinical effects on patients. Although several therapeutic drugs have been developed to treat acne, they cannot provide a radical cure and may even cause serious side effects. Better therapeutic agents are therefore needed for the treatment of this condition [40].

FGF21 has been reported to be effective in the treatment of LPS-induced acute lung injury by affecting inflammation and apoptosis. In addition, FGF21 has been found to downregulate the expression of the doxorubicin-induced inflammatory cytokines TNF-α and IL-6 through the IKK/IκBα/nuclear factor-κB pathway, showing that FGF21 has anti-inflammatory activity in cardiac dysfunction and pathological changes [41,42,43]. These findings suggest that FGF21 may also have anti-inflammatory effects on inflammation induced by *C. acnes*. *C. acnes* injection significantly increased the expression of FGF21 in mouse ears, with immunohistochemical staining showing that most of the FGF21 was expressed in the dermis (Figure 1A,B). Products of the KLB gene act as FGFR coreceptors in the endocrine FGFR subfamily, with the FGF–FGFR–KLB axis involved in regulating fibrosis and fat infiltration in fibrous and fatty diseases [44]. Assays of the expression of FGFR1 and KLB in mouse tissues showed that most of these proteins were concentrated in the epidermis (Figure 1C–F). These findings suggest that FGF21 may act on the skin in a paracrine manner to reduce the inflammation induced by *C. acnes*. A co-culture of NHDFs and NHEKs showed that, following stimulation with *C. acnes* in vitro, the secretion of FGF21 by NHDF cells was markedly increased, significantly reducing the expression of *C. acnes*-induced proinflammatory factors in NHEK cells, with these changes counteracted by the administration of FGF21-neutralizing antibody (Figure 2B–D). The anti-inflammatory effects of FGF21 were reversed by blocking FGFR1 or KLB, which confirmed the importance of the binding of FGF21 to receptor complexes of FGFR1 and KLB (Figure 2E,F and Figure S2D,E). FGF21 has been reported to act in a paracrine fashion to promote cell migration and differentiation during wound healing and to act in a dual paracrine fashion to increase KGF expression, promoting fibroblast activation in skin fibrosis. These findings are consistent with the mechanism of action of FGF21 observed in this study [45,46]. FGF21 is mainly secreted by the liver and adipose tissue, with these tissues being important sources of FGF21 in some diseases [47]. Generally, the present study tends to probe the target effects of FGF21 rather than its sources. To the best of our knowledge, the present study is the first one to show that FGF21 regulates and plays an important role in the inflammation induced by *C. acnes* through interactions between the dermis and the epidermis. However, more detailed studies are still needed to define cell–cell interactions and signaling mechanisms associated with FGF21.

**Figure 8:**
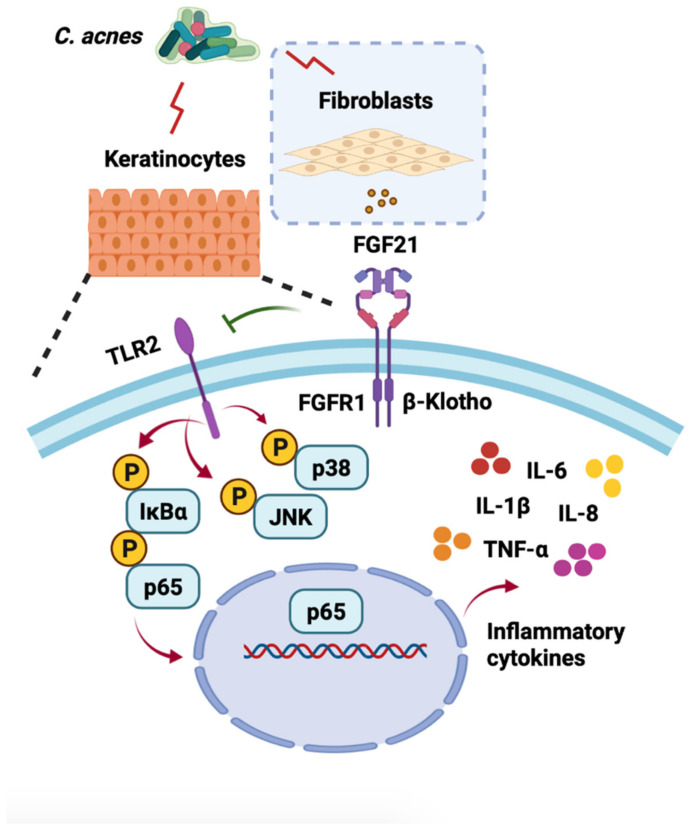
Proposed mechanism of action of FGF21 in *C. acnes*-induced skin disease. Under the induction of *C. acnes*, FGF21 secreted from fibroblasts inhibits TLR2, NF-KB, and MAPK signaling and inflammatory factors in keratinocytes by activating FGFR1 and KLB, and exogenous treatment by FGF21 can also relieve a series of inflammatory reactions induced by *C. acnes*.

Acne may be relieved by the administration of several exogenous drugs. Some small peptides, such as melittin and short lipopeptides, can protect cells from inflammatory responses induced by *C. acnes* and may even have direct antibacterial effects [48]. Several biologically active compounds extracted from plants have been found to inhibit the occurrence of acne to varying degrees [49,50,51,52]. The present study, showing that endogenous FGF21 can suppress the inflammation induced by *C. acnes*, suggests that the administration of exogenous FGF21 can be used to prevent inflammation caused by *C. acnes* invasion. The transcription factor NF-κB plays an indispensable role in regulating the immediate transcription response in inflammatory conditions. NF-κB activation is closely related to the pathogen-recognizing receptors, including TLRs [53,54], and many core proteins in the NF-κB pathway are involved in the development of innate immune responses [55,56]. The effects of FGF21 treatment on the levels of expression of p65 and IκB (Figure 3A–C and Figure 6A–C) and on other signal cascades related to inflammation and MAPK signaling, including JNK and p38 (Figure 3D and Figure 6D), were therefore investigated. In addition, FGF21 alleviated the redness and swelling in mouse ears and effectively reduced the expression of inflammatory cytokines in vivo and in vitro (Figure 3E and Figure 5D). These anti-inflammatory properties of FGF21 suggest a possible mechanism in acne (Figure 8). Testing also showed that FGF21 is nontoxic in vivo and in vitro (Figure S4A–D).

Other cells may also be involved in the pathogenesis of acne and in the effects of FGF21. Knowledge of these novel physiological functions of FGF21 may aid in the development of new treatment options to combat inflammation induced by *C. acnes*.

## 4. Materials and Methods

### 4.1. Bacterial Strain Culture

*C. acnes* (ATCC6919) was used throughout this study. It was cultured at 37 °C in the Brain Heart Infusion Broth medium under anaerobic conditions until it reached OD600 = 1.0 (logarithmic growth phase). The bacterial suspension was centrifuged at 3000× *g* for 10 min at 4 °C. To stimulate cells, *C. acnes* was heat inactivated at 80 °C for 30 min and serially diluted to different concentrations. For intradermal injection in mouse ears, living *C. acnes* was directly diluted to the indicated concentrations.

### 4.2. Cell Culture and Stimulation

The HaCaT cells (Procell, CL-0090, Wuhan, China) were obtained from Procell in Wuhan, China, and cultured in MEM medium (Procell, PM150410, Wuhan, China) supplemented with 10% FBS and 1% penicillin/streptomycin at 37 °C in a humidified atmosphere of 5% CO_2_. Before reagent treatment, the cells were cultured in MEM medium for starvation overnight. The cells grew to 80% confluence before they were stimulated with a specified dose of heat-inactivated *C. acnes* (1.0 × 10^7^ CFU/mL), FGF21 (20 ng/mL, dissolved in PBS and produced by Wenzhou Medical University Gene Engineering Laboratory, Wenzhou, China), or different inhibitors (10 mM Bay11082, MCE; 20 mM SP600125, Selleck; 10 mM SB203580, MCE, NJ, USA). Then the cells were collected at specified times for RNA isolation, Western blotting, or immunofluorescence and the supernatants were harvested for an enzyme-linked immunosorbent assay (ELISA, Beyotime Biotechnology, PF313).

### 4.3. Extraction and Culture of Human Primary Epidermal Keratinocytes (NHEKs) and Human Primary Dermal Fibroblasts (NHDFs)

NHEKs and NHDFs were obtained from prepuce tissue (children aged 6–8) at the Second Affiliated Hospital of Wenzhou Medical University. The separation and extraction techniques have been reported previously [57,58]. Briefly, after removing fat and subcutaneous tissue from 2% penicillin/streptomycin PBS, epidermal–dermal separation was performed using a 0.05% dispase II neutral protease (Sigma Aldric, St. Louis, MO, USA, D4693-1G) solution overnight at 4 °C. Then epidermis and dermis were quickly separated and washed with cold PBS. To establish the primary keratinocyte culture, the epidermal slices were incubated in 0.25% trypsin (Gibco, 25200072) at 37 °C until the epidermal slice started to disaggregate into single cells. Afterward, the cells were filtered through a cell filter with 70 μm wells to obtain a single cell suspension and then centrifuged to obtain cells by re-suspension. Finally, the cells were placed on six-well plates precoated with a growth-factor-reduced matrix (Corning, New York, NY, USA, 354234) and cultured in a serum-free keratinocyte medium (Sigma Aldric, 133-500). To establish the primary fibroblast culture, the dermis was shredded and placed in 25 cm^2^ FBS-coated culture flasks and placed horizontally in the incubator for 1 h and then vertically for 3 h. What is more, the tissues were cultured in DMEM (Gibco, 11054020) supplemented with 5.5 mM glucose, 10% fetal bovine serum, and 1% penicillin/streptomycin, followed by medium changes every 3 days.

Written informed consent was obtained from all subject in this study, which was conducted according to the ethical standards of Wenzhou Medical University.

### 4.4. Co-Culture System

The cells were divided into two compartments using a Transwell insert (Coring, 3413). Keratinocytes and fibroblasts were placed in the upper and lower compartments, respectively, and cultured for a period of time and *C. acnes* was added. After 6 h, FGF21-neutralizing antibody (MAB25373, R&D Systems, Minneapolis, MN, USA) (4 μg/mL) or control goat IgG (AB108-C, R&D Systems) was added to the culture medium for further study.

### 4.5. Animal Model

Wild-type (WT) C57 BL/6J mice were obtained from the Model Animal Research Center of Nanjing University and were housed 4 per cage, given commercial chow and tap water, and maintained at 22 °C on a 12 h/12 h light/dark cycle.

The 6–8-week-old C57 BL/6J mice were randomly divided into different groups and maintained under various conditions. The mouse ears of the model group were intradermally injected with *C. acnes* in PBS at the concentration of 1.0 × 10^7^ CFU in 20 μL. For the treatment group, the *C. acnes* suspension (1.0 × 10^7^ CFU/20 μL) and FGF21 (50 μg/mL) were injected into the mouse ears. At the same time, equivalent amounts of PBS were injected into the mouse ears as a control. At the end of each treatment period (24 h later), the animals were subjected to general anesthesia and the ears were removed for follow-up treatment.

All experimental procedures and methods were approved by the Institutional Animal Care and Use Committee of Wenzhou Medical University.

### 4.6. Western Blot Analysis

RIPA lysis buffer (Thermo Fisher Scientific, Waltham, MA, USA) with a protease and phosphatase inhibitor cocktail (Abcam, Cambridge, MA, USA) was used to extract total proteins from fresh mouse ears and cell samples. The nuclear proteins were separated using Pierce NE-PER Nuclear and Cytoplasm Extraction Kit (Thermo Fisher Scientific). The protein concentration was measured using Pierce BCA Protein Assay Kit (Thermo Fisher Scientific). Briefly, 30 μg of protein was separated by sodium dodecyl sulfate–polyacrylamide gel electrophoresis (SDS–PAGE), transferred to polyvinylidene difluoride (PVDF) (Millipore, Burlington, MA, USA), and then blocked with 5% skim milk (BD Bio-sciences, San Jose, CA, USA). After incubation with the primary and secondary antibodies, each blot was developed using ECL reagent (Millipore) and captured by the Amersham Image 600 system (GE Healthcare Life Sciences, Pittsburgh, PA, USA). The protein levels were normalized to GAPDH expression. Primary antibodies in the Western blot are listed in Supplementary Table S1.

### 4.7. RNA Extraction and qRT-PCR

RNA extraction was performed with TRIzol Reagent (Invitrogen, Waltham, MA, USA) according to the manufacturer’s recommendations. The complementary DNA was reverse transcribed using the Vazyme (Nanjing, China) reverse transcription system, and a qRT-PCR was performed with SYBR Green PCR Master Mix (Vazyme) according to the manufacturer’s protocol. The mRNA levels were normalized to GAPDH expression. Primary-gene-specific primer sequences are shown in Supplementary Table S2.

### 4.8. RNA Interference

For RNA interference, the HaCaT cells were transfected with control scramble siRNA (Santa Cruz Biotechnology, Dallas, TX, USA) and FGFR1 or KLB siRNA (Santa Cruz Biotechnology). Lipofectamine 2000 transfection reagent (Thermo Fisher Scientific) was first transfected into Opti-MEM (Gibco) for 6–8 h, and then MEM medium was changed for another 12 h, followed by subsequent experiments.

### 4.9. Histology, Immunohistochemistry (IHC), and Immunofluorescence Staining

Paraffin-embedded tissue sections (7 mm thick) were installed on glass slides for hematoxylin and eosin (H&E) staining in order to evaluate ear swelling and inflammatory infiltration in mice. After dewaxing, the slides were stained with the H&E staining kit (Solarbio, Beijing, China) as per the manufacturer’s protocol.

Immunohistochemistry and immunofluorescence were performed after antigen repair, followed by 0.5% triton-100 and 5% bovine serum albumin (BSA) at room temperature for 15 min and 30 min, respectively. Next, the primary antibody was incubated overnight at 4 °C and the secondary antibody was added for incubation. The dilution ratio was operated according to the reagent specification. For the IHC staining, DAB Horseradish Peroxidase Color Development Kit (Beyotime, Shanghai, China) was used, followed by reverse staining with hematoxylin. For immunofluorescence, the cell nuclei were stained with DAPI. The images of H&E and IHC staining were captured by using a Nikon ECLIPSE Ni microscope. The immunofluorescence images were visualized and captured by using a Leica SP8 confocal microscope after incubation with primary antibodies of FGF21 (Abcam, AB171941), FGFR1 (CST, 9740S), β-Klotho (Abcam, AB33416), p65 (Proteintech, 66535-1-IG), MPO (Santa Cruz, SC-390109), CD68 (ABclonal, Woburn, MA, USA, A13286), TLR2 (ABclonal, A19125), and CD11b (ABclonal, A1581).

### 4.10. Statistical Analysis

All data in this study were analyzed by GraphPad Prism 8.0 and were presented as the mean ± the SD. The statistics were compared and analyzed by unpaired two-tailed Student’s *t*-test (between two experimental groups) or one-way ANOVA (between numerous experimental groups). The criterion *p* < 0.05 was used to determine statistical significance.

## 5. Conclusions

The findings of this study indicate that FGF21 in the dermis cells inhibits epidermal *C. acnes*-induced inflammation in a paracrine manner, with FGF21 performing dermal-epidermal interaction through FGFR1 and KLB. In addition, FGF21 treatment suppresses the *C. acnes*-induced activation of the TLR2/NF-κB/MAPK signaling pathways and the secretion of inflammatory cytokines in vitro and in vivo.

## Data Availability

The data presented in this study are available on request from the corresponding author.

## Supplementary Materials

The following supporting information can be downloaded at https://www.mdpi.com/article/10.3390/ijms23073589/s1.
