# Supplementary material for: Suppression of Cutibacterium acnes-Mediated Inflammatory Reactions by Fibroblast Growth Factor 21 in Skin
Source: Int J Mol Sci. 2022 Mar 25;23(7):3589. doi: 10.3390/ijms23073589 (PMC8998725; doi:10.3390/ijms23073589)
Supplement: Supplementary file 1 [file ijms-23-03589-s001.zip › External supplementary.pdf]

Table S1. A list of abbreviations in this study.

Abbreviations	Full Names
C. acnes	*Cutibacterium acnes*
FGF21	Fibroblast growth factor 21
FGFR1	Fibroblast growth factor receptor 1
KLB	β-Klotho
TLR2	Toll-like receptor 2
NF-κB	Nuclear factor-κB
MAPK	Mitogen-activated protein kinase
IL-1β/6/8	Interleukin (IL)-1β/6/8
TNF-α	Tumor necrosis factor-α
FBS	Fetal bovine serum
NHDF	Normal human dermal fibroblasts
NHEK	Normal human epidermal keratinocytes

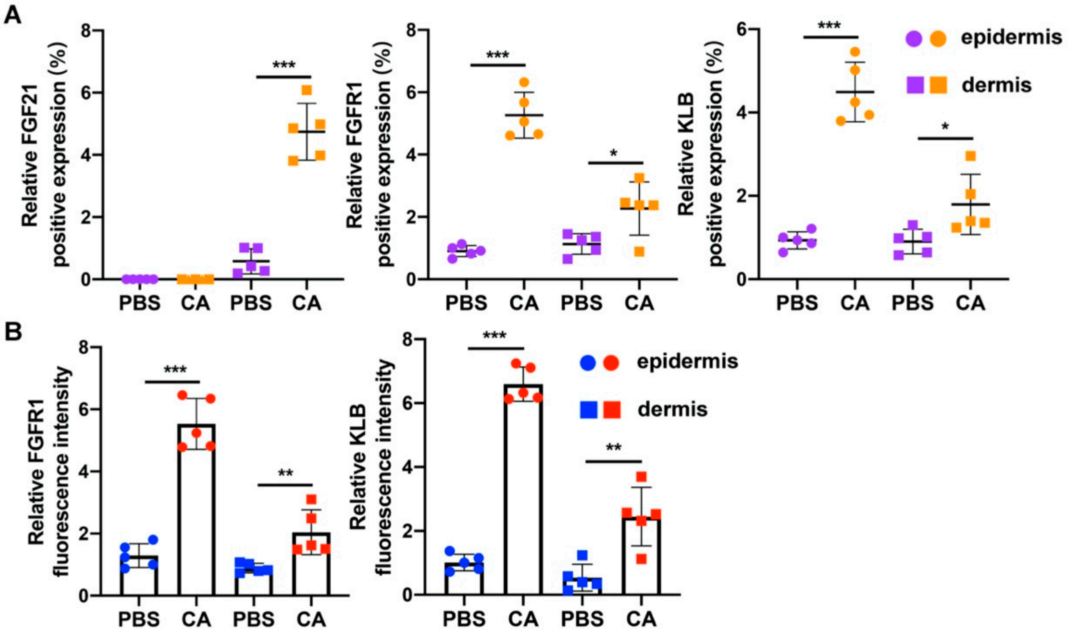

Figure S1. Immunohistochemistry (IHC) and immunofluorescence staining of FGF21, FGFR1, KLB in mouse ears.

(A) Analysis of FGF21, FGFR1 and KLB positive expression in the epidermis and dermis in ears of PBS or *C. acnes*-induced mice (n = 5 mice/group). (B) Analysis of FGFR1 and KLB fluorescence intensity in the epidermis and dermis in ears of PBS or *C. acnes*-induced mice (n = 5 mice/group). All data are presented as mean ± SD, $*P < 0.05$, $**P < 0.01$, $***P < 0.001$.

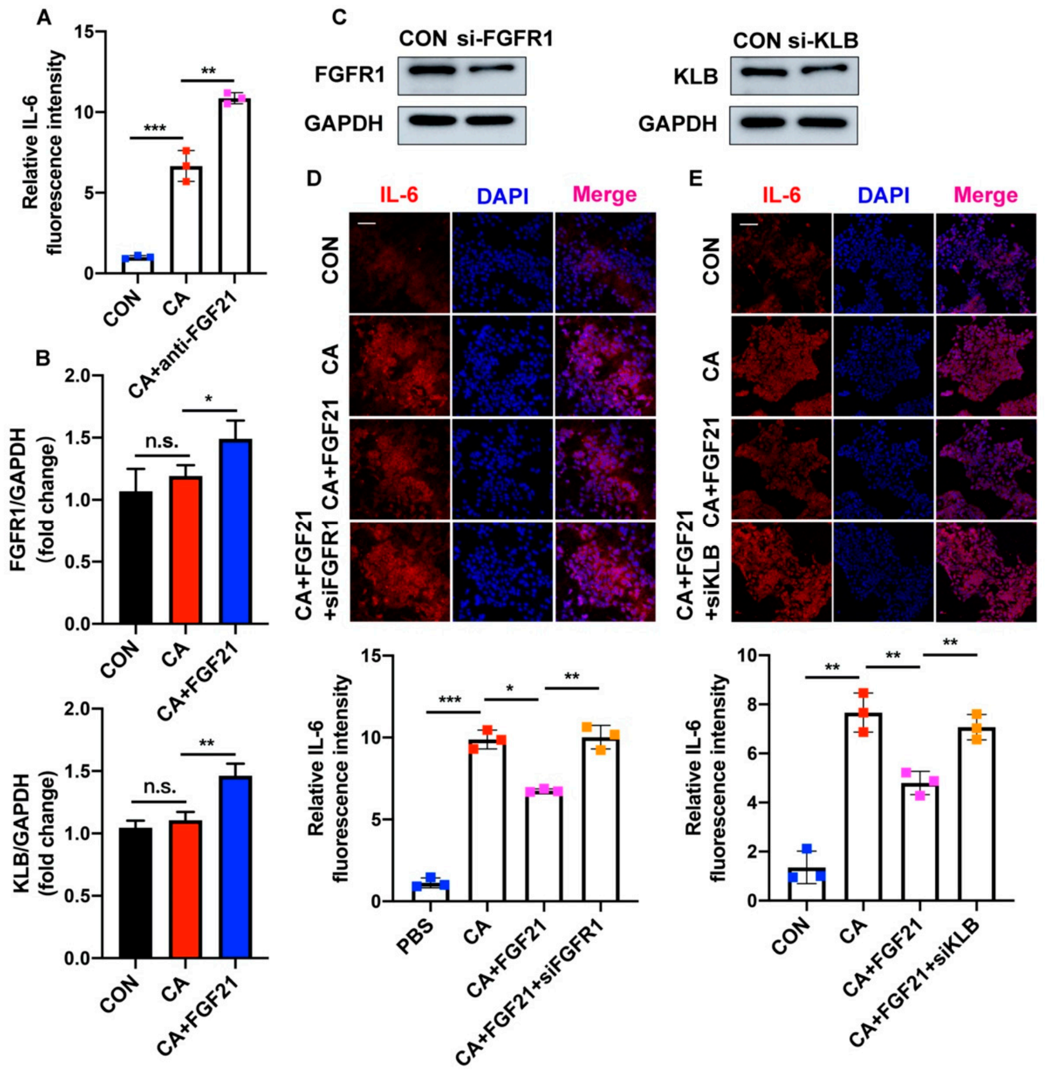

Figure S2. Blocking of FGF21, FGFR1 and KLB increased the expression of proinflammatory cytokines.

(A) Immunofluorescence staining of IL-6 in the fibroblast-keratinocyte co-culture system. (B) Quantification of FGFR1 and KLB at protein levels. (C) Protein expression levels of FGFR1 and KLB in FGFR1-specific or KLB-specific siRNA interference HaCat cells. (D, E) Representative confocal scans were shown for IL-6 (red) after transfection of FGFR1 and KLB small interfering RNA, nuclei were stained with DAPI (blue). Scale bars = 100 μm. All data are presented as mean ± SD, *$P < 0.05$, **$P < 0.01$, ***$P < 0.001$, n.s., non-significant.

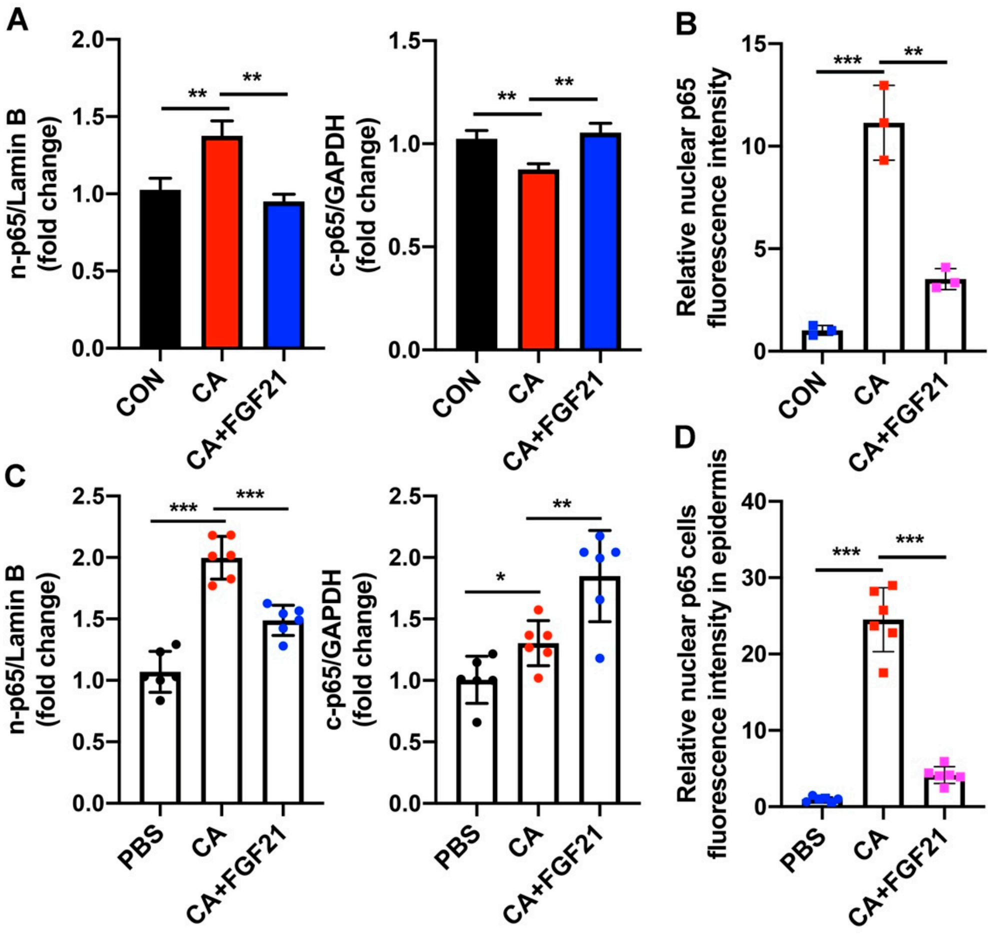

Figure S3. FGF21 inhibits *C. acnes*-induced translocation of p65 into the nucleus.

Quantification of the p65 from HaCaT cells (A) and ear tissue (C) in cytoplasmic and nuclear extracts. The statistics of nuclear p65 fluorescence intensity in HaCaT cells (B) and mice ears (D). All data are presented as mean ± SD, **P* < 0.05, ***P* < 0.01, ****P* < 0.001.

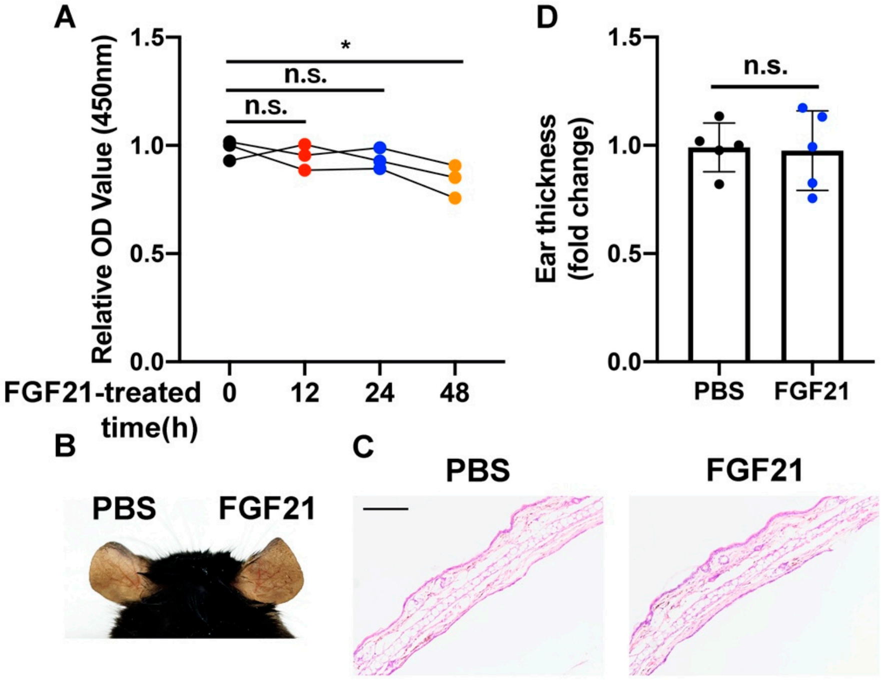

Figure S4. Cytotoxic effects of FGF21 in HaCaT cells and mouse ears.

(A) Cytotoxicity of FGF21 was determined by CCK-8 assay in HaCaT cells. The HaCaT cells were treated with 20 ng/mL of FGF21 for 6, 12, and 24 h. (B) The image of mice ears subjected to inject PBS or FGF21 (50 μg/mL). (C, D) Histological changes and ear thickness were measured by H&E staining. All data are presented as mean ± SD, *$P < 0.05$, n.s., non-significant.
